# Potential role of salivary lactic acid bacteria in pathogenesis of oral lichen planus

**DOI:** 10.1186/s12866-024-03350-0

**Published:** 2024-06-07

**Authors:** Xiaomeng Ren, Dan Li, Mimi Zhou, Hong Hua, Chunlei Li

**Affiliations:** 1 Department of Oral Medicine, Peking University School and Hospital of Stomatology & National Center of Stomatology & National Clinical Research Center for Oral Diseases & National Engineering Research Center of Oral Biomaterials and Digital Medical Devices, 22 Zhongguancun Avenue South, Haidian District, Beijing, 100081 PR China; 2 Department of Stomatology, Xiongan Xuanwu Hospital, Baoding, Hebei PR China; 3 Key Laboratory of Oral Biomedical Research of Zhejiang Province, The Affiliated Hospital of Stomatology, School of Stomatology, Department of Oral Medicine, Zhejiang University School of Medicine, Hangzhou, Zhejiang PR China

**Keywords:** Oral lichen planus, Oral microbiota, Lactic acid bacteria, *Lactococcus*, *Lactococcus lactis*

## Abstract

**Background:**

Emerging evidence emphasized the role of oral microbiome in oral lichen planus (OLP). To date, no dominant pathogenic bacteria have been identified consistently. It is noteworthy that a decreased abundance of *Streptococcus*, a member of lactic acid bacteria (LAB) in OLP patients has been commonly reported, indicating its possible effect on OLP. This study aims to investigate the composition of LAB genera in OLP patients by high-throughput sequencing, and to explore the possible relationship between them.

**Methods:**

We collected saliva samples from patients with OLP (*n* = 21) and healthy controls (*n* = 22) and performed 16 S rRNA gene high-throughput sequencing. In addition, the abundance of LAB genera was comprehensively analyzed and compared between OLP and HC group. To verify the expression of *Lactococcus lactis*, real time PCR was conducted in buccal mucosa swab from another 14 patients with OLP and 10 HC. Furthermore, the correlation was conducted between clinical severity of OLP and LAB.

**Results:**

OLP and HC groups showed similar community richness and diversity. The members of LAB, *Lactococcus* and *Lactococcus lactis* significantly decreased in saliva of OLP cases and negatively associated with OLP severity. In addition, *Lactococcus* and *Lactococcus lactis* showed negative relationship with *Fusobacterium* and *Aggregatibacter*, which were considered as potential pathogens of OLP. Similarly, compared with healthy controls, the amount of *Lactococcus lactis* in mucosa lesion of OLP patients was significantly decreased.

**Conclusions:**

A lower amount of *Lactococcus* at genus level, *Lactococcus lactis* at species level was observed in OLP cases and associated with disease severity. Further studies to verify the relationship between LAB and OLP, as well as to explore the precise mechanism is needed.

**Supplementary Information:**

The online version contains supplementary material available at 10.1186/s12866-024-03350-0.

## Background

Oral lichen planus (OLP) is a common inflammatory disease, which affects approximately 1.5% of the general population and is prevalent among middle-aged and elderly women [1]. It is classified as an oral potentially malignant disorder, and the malignant transformation rate is 0.44–2.28% [2]. Although OLP has been extensively studied, its etiopathogenesis is unclear. OLP is a multifactorial disease, with genetic predisposition, psychological factors, immune dysregulation, and microbial infection being the potential triggers [3, 4]. Previous studies have reported that the structure and composition of oral microbiota changes in OLP and could be related to the progression of the disease [5, 6]. Human microbiome has been related to host health. The oral microbiota is the second largest bacteria community of the body, comprising mostly commensal bacteria. Under certain conditions, oral microbiota undergoes compositional and/or functional alterations (dysbiosis) that lead to inflammation and abnormal immune response, contributing to several local and systemic diseases [7]. Previous studies have reported oral microbial dysbiosis in OLP patients, and several microorganisms have been identified to be associated with disease progression, including *Prevotella* and *Fusobacterium* [8–10]. Although the possible role of microbiome in OLP has been reported, same dominant pathogenic bacteria have not been identified in previous studies. Several studies have reported the decreased abundance of *Streptococcus* [10–13], a member of lactic acid bacteria (LAB), in OLP cases, indicating the close relationship between them. Moreover, LAB inhibit the growth of uropathogens, enteropathogens, and oral pathogens, but their infection-fighting mechanism is complex and unclear [14, 15]. However, the potential effect of friendly microbes, such as LAB, on the pathogenesis of OLP and their correlation with OLP disease severity has not been elucidated. Most previous studies have investigated the difference in highly abundant flora between OLP and control population; therefore, the clinical value and mechanisms of potential disease-related microbes with low abundance may have been overlooked.

In the present study, we investigated the difference in the oral microbiota composition between OLP patients and healthy control (HC) participants, with a focus on the abundance of LAB.

## Methods

### Participants

This study was approved by the Peking University Institutional Review Board, China [PKUSSIRB 202161004]. Participants were enrolled in Department of Oral Medicine, Peking University School and Hospital of Stomatology, China. We have followed the guidelines of the Helsinki Declaration in this investigation.

#### Inclusion criteria of patients with OLP

(1) age: 18–65 years; (2) number of natural teeth remaining: ≥20; and (3) OLP patients who were clinically and histologically diagnosed according to the World Health Organization (WHO) criteria (2003) [16].

The HC group included individuals without oral mucosa diseases.

#### Participant exclusion criteria

(1) pregnancy or lactation; (2) other known oral mucosal diseases; (3) life-threatening systemic diseases or autoimmune diseases; (4) use of immunomodulator or antibiotic within the 1-month-period before the start of the study; (5) use of any mouthwash within the 7-day-period before the start of the study; (6) tobacco or alcohol use; (7) severe periodontitis (clinical attachment loss: ≥5 mm, probing depth (PD): >6 mm, and extension of bone loss to the apical portion of the root), visible caries, and dentures.

The present study had two cohorts. We collected 43 saliva samples from patients with OLP (*n* = 21) and HC individuals (*n* = 22) and performed 16 S rRNA gene high-throughput sequencing in cohort 1. Cohort 2 included 24 swab samples of normal buccal mucosa (*n* = 10) and OLP buccal mucosa lesion (*n* = 14) to detect the amount of *Lactococcus lactis*. Clinical information on 2 cohorts was presented in Table 1.

**Table 1:** General information of HC and OLP participants

	HC group	OLP group	*P* value
Cohort 1 (saliva for 16 S rRNA sequencing)			
Age (year)*	49.05 ± 9.10	43.86 ± 11.08	0.100
Male/Female	2/20(*n* = 22)	5/16(*n* = 21)	0.372
PD (mm)*	2.95 ± 0.23	2.89 ± 0.25	0.489
Cohort 2 (swab for real time PCR)			
Age (year)*	45.50 ± 12.10	50.79 ± 12.53	0.313
Male/Female	2/8(*n* = 10)	5/9(*n* = 14)	0.704

*Mean ± SD; HC: healthy control; OLP: oral lichen planus; PD: probing depth; PCR: polymerase chain reaction

### Clinical examination

The severity of OLP lesions was evaluated using the reticular/hyperkeratotic, erosive/erythematous, ulcerative (REU) scoring system, as reported in a previous study [17]. Briefly, the scores were assigned based on the examination of reticular/hyperkeratotic (R) (0: none; 1: present), erythematous (E) and/or ulcerative (U) lesions (0: none; 1: lesions < 1 cm^2^; 2: lesions ranging in size from 1 to 3 cm^2^; 3: lesions > 3 cm^2^), and the total REU score was calculated as follows: REU = ∑ (R + E × 1.5 + U × 2.0).

### Sample collection

All participants were instructed to avoid drinking or eating for 2 h before sampling. Samples were obtained between 8:00 to 11:00 AM. We collected 5 mL of whole unstimulated saliva in a sterile conical tube from each participant in cohort 1 using standard techniques. The tube containing saliva was centrifuged at 12,000 g for 15 min, the supernatant was removed and the precipitate was kept. Samples of cohort 2 were obtained by rotating a swab pressed to the buccal mucosa. All samples were stored at − 80 °C for further analyses [18].

### DNA extraction, amplicon generation for sequencing

Total DNA from each sample in cohort 1 was extracted using the cetyltrimethyl ammonium bromide method [19]. DNA concentration and purity were evaluated on 1% agarose gels. After quantitation, DNA samples were diluted using sterile water to a final concentration of 1 ng/µL. The V3–V4 region of the 16 S rRNA gene was amplified by polymerase chain reaction (PCR) from the diluted DNA samples using the bacterial universal primers 343 F (5’-TACGGRAGGCAGCAG-3’) and 798R (5’-AGGGTATCTAATCCT-3’) in a T100PCR (BioRad, Hercules, CA, USA). Next, the obtained PCR products were mixed and purified using the Qiagen Gel Extraction Kit (Qiagen, Hilden, Germany).

### Relative expression of *Lactococcus lactis* in HC and OLP by real-time PCR

Total bacteria DNA was extracted from mucosa swab using TIANamp Bacteria DNA Kit (DP302, TianGen Biotech, Beijing, China). And the quantitative PCR was performed using Universal SYBR Green Fast qPCR Mix (RK21203, ABclonal, Wuhan, China) following the protocol. Primers were: 16S rRNA universal: (F)5’- CGCTAGTAATCGTGGATCAGAATG-3’ and (R) 5’-TGTGACGGGCGGTGTGTA-3’ [20]; *Lactococcus lactis*: (F)5’- TGTCACAAGCCATGCGTAAAC − 3’ and (R)5’- CACGCAATTGGTTGATGAAAA − 3’ [21]. The expression level of *Lactococcus lactis* were normalized to 16 S rRNA universal and were calculated using 2^−∆∆Ct^ method.

### Sequence and data analysis

Sequencing libraries were generated using the TruSeq® DNA PCR-Free Sample Preparation Kit (Illumina, San Diego, CA, USA) according to the manufacturer’s instructions, and index codes were added. The quality of the library was assessed using a Qubit@ 2.0 fluorometer (Life Technologies, Carlsbad, CA, USA). Finally, the PCR products were sequenced and analyzed using the Novaseq 6000 platform (Illumina, San Diego, CA, USA) according to the manufacturer’s instructions.

The sequencing reads were assigned to each sample according to their unique barcode. Paired-end reads were preprocessed using the Cutadapt software to detect and cut off the adapter. After trimming paired-end reads, low quality sequences were filtered, denoised, merged, and chimera reads were detected and cut off using DADA2 with the default parameters of the QIIME 2 platform [22, 23]; amplicon sequence variant (ASV) abundance table was obtained as the output.

The representative read of each ASV was selected using the QIIME 2 package. All representative reads were annotated and blasted against the SILVA database using the q2-feature-classifier with default parameters. The microbial diversity in WUS samples was estimated using the alpha diversity indexes, namely Chao1 and Shannon indexes, which measure species richness and species diversity, respectively, in a sample. The Binary Jacard algorithm run in the QIIME platform was used for UniFrac principal coordinates analysis (PCoA). A linear discriminant analysis effect size (LefSe) algorithm was used to identify potential biomarkers of OLP, and the linear discriminant analysis (LDA) threshold was set as 3.

### Statistical analyses

Data were analyzed using the SPSS version 26.0 statistical package (SPSS® Inc., Chicago, IL, USA). Graphs were prepared using the software GraphPad Prism 9 (GraphPad Software, San Diego, CA, USA). Normality and homogeneity of variance were evaluated. Categorical data were analyzed using the Chi-square test or Fisher’s exact test for different groups. Continuous data were presented as mean ± standard deviation (SD), and independent-samples *t*-test or nonparametric test was used to analyze difference in data between two groups. Correlation analysis was performed using Spearman’s correlation coefficient. Differences were considered significant at *P* < 0.05.

## Results

### Sequence data

A total of 2,649,963 merged sequenced reads were obtained from all samples of both groups. The clean tags ranged between 35,446 and 71,753 reads after quality control. After removing chimera sequences, the valid tags ranged between 29,387 and 68,865 reads, with an average of 61,627 sequences for each sample. Finally, 120 to 1048 ASVs were identified. OLP and HC groups shared 981 ASVs. Moreover, the rarefaction curve tended to be flat, indicating that the 16 S rRNA gene sequences identified in this study represented the majority of the bacteria present in saliva samples.

### Species richness and diversity of oral microbiota in OLP and HC groups

**Fig. 1:**
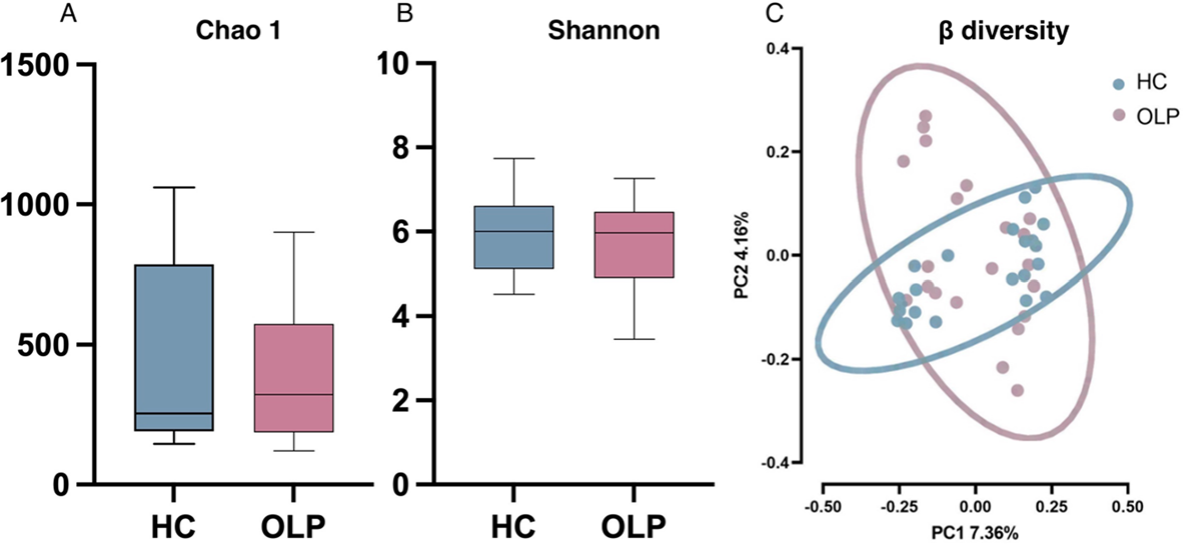
Alpha and Beta diversity analysis of microbiota in saliva samples of OLP and HC groups. (**A**) Chao 1 (**B**) Shannon index of diversity. *P* > 0.05; (**C**)Principal coordinates analysis (PCoA) plot constructed using the Binary Jaccard algorithm. *P* = 0.041

Alpha diversity analysis, based on Chao 1 and Shannon indexes, did not reveal significant differences in species richness and diversity, respectively, of oral microbiota between OLP and HC groups (*P* > 0.05; Fig. 1A, B).

The PCoA plot revealed obvious separation between HC and OLP groups, indicating that the overall structure of the bacterial community in the two groups was significantly different (*P* = 0.041). Moreover, the OLP patients exhibited distinct discrete characteristics, indicating extensive heterogeneity (Fig. 1C). PC1 explained 7.36% variability, whereas PC2 explained 4.16% variability.

### Phylum- and genus-level identification of saliva microbiota in OLP and HC groups

All operational taxonomic units obtained from both groups were clustered into 9714 ASVs, representing 42 phyla, 105 classes, 243 orders, 390 families, and 755 genera.

**Fig. 2:**
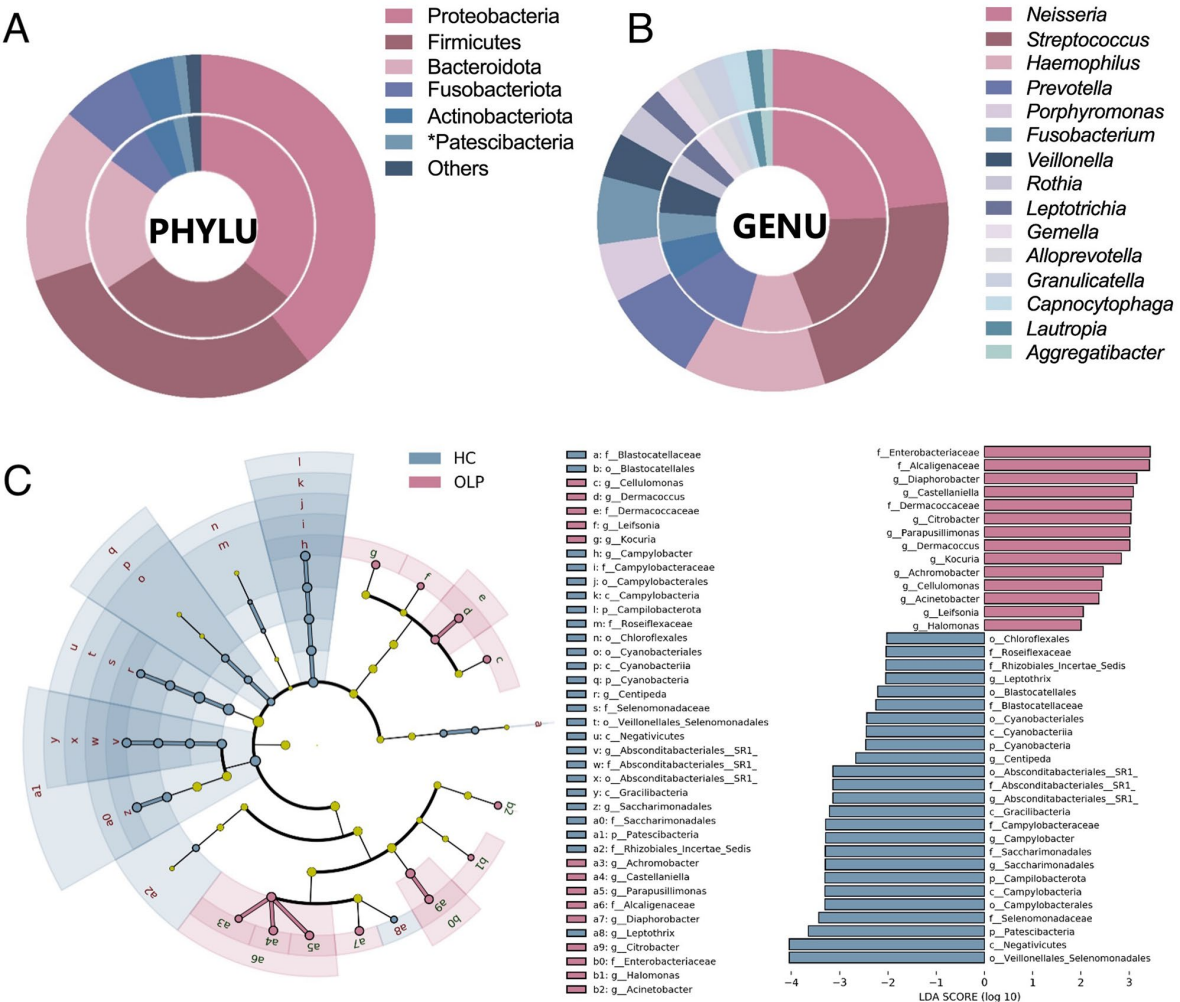
Analysis of relative abundance of microbiota in saliva sample of HC and OLP groups. Donut chart of main communities at (**A**) phylum and (**B**) genus levels in HC (inner ring) and OLP (outer ring). *P < 0.05; (**C**) LDA (threshold was set at 3) using LefSe algorithm

At the phylum level, 98% sequences belonged to Proteobacteria, Firmicutes, Bacteroidota, Fusobacteria, Actinobacteria, and Patescibacteria in both OLP and HC groups. Moreover, Patescibacteria exhibited higher abundance in the HC group than in the OLP group (Fig. 2A).

At genus level, the relative abundance of 15 genera was > 1% in both OLP and HC groups, with *Neisseria* and *Streptococcus* accounting for approximately 20%, and *Haemophilus* and *Prevotella* accounting for approximately 10%. The abundance between two groups showed no significant difference (Fig. 2B).

LDA using LefSe revealed differences between OLP and HC groups at different taxonomic levels, including 3 phyla, 4 classes, 6 orders, 10 families. and 16 genera. Moreover, compared with those in the HC group, the abundance of Patescibacteria (phylum level), Gracilibacteria (class level), and Absconditabacteriales_SR1 (order, family, and genus levels) was significantly decreased in the OLP group, whereas that of *Achromobacter* and *Citrobacter* at the genus level was significantly increased (Fig. 2C).

### Abundance of LAB in OLP and HC groups

**Fig. 3:**
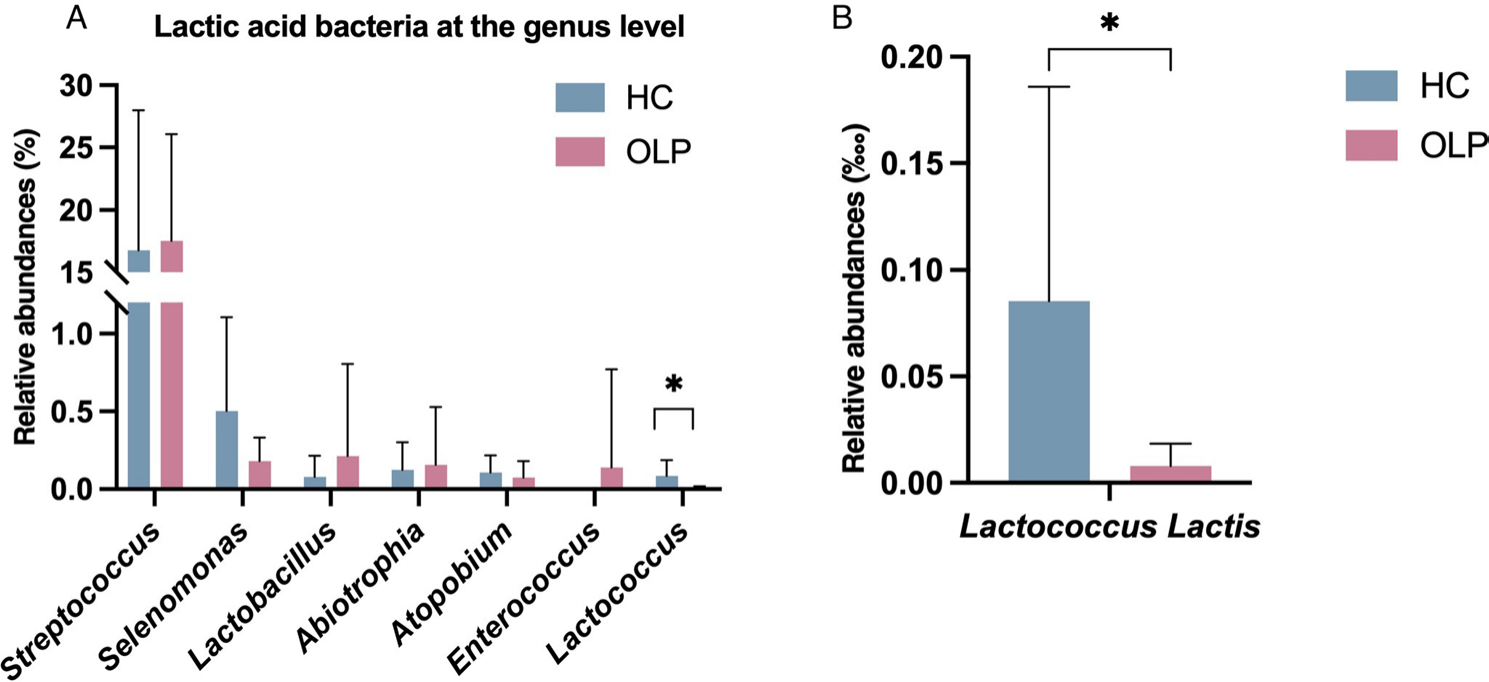
Relative abundance of LAB in saliva sample of HC and OLP groups. (**A**) lactic acid bacteria at the genus level and (**B**) *Lactococcus lactis* in HC and OLP groups. *P < 0.05

**Fig. 4:**
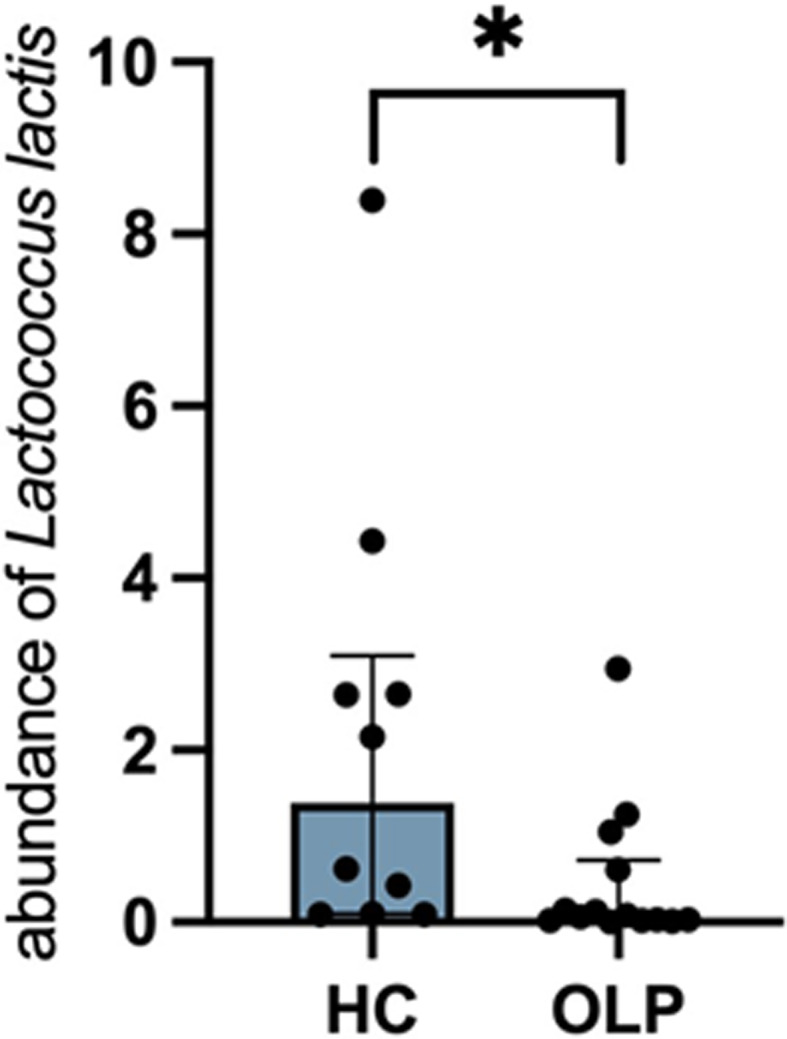
Relative quantification of *Lactococcus lactis* in swab sample of HC and OLP groups. *P < 0.05

Next, we investigated the composition of LAB at the genus level in OLP and HC groups. The abundance of *Streptococcus*, *Selenomonas*, *Lactobacillus*, *Abiotrophia*, and *Enterococcus* did not exhibit significant differences between OLP and HC groups, whereas the abundance of *Lactococcus* was significantly lower in the OLP group than in the HC group. Moreover, the abundance of *Lactococcus lactis* was significantly decreased in OLP patients compared with that in HC participants (Fig. 3). The real-time PCR of swab sample in cohort 2 further validated that *Lactococcus lactis* presented lower amount in patients with OLP rather than HC with statistically significance (Fig. 4).

### Potential impact of LAB on microbiome composition shift in OLP

**Fig. 5:**
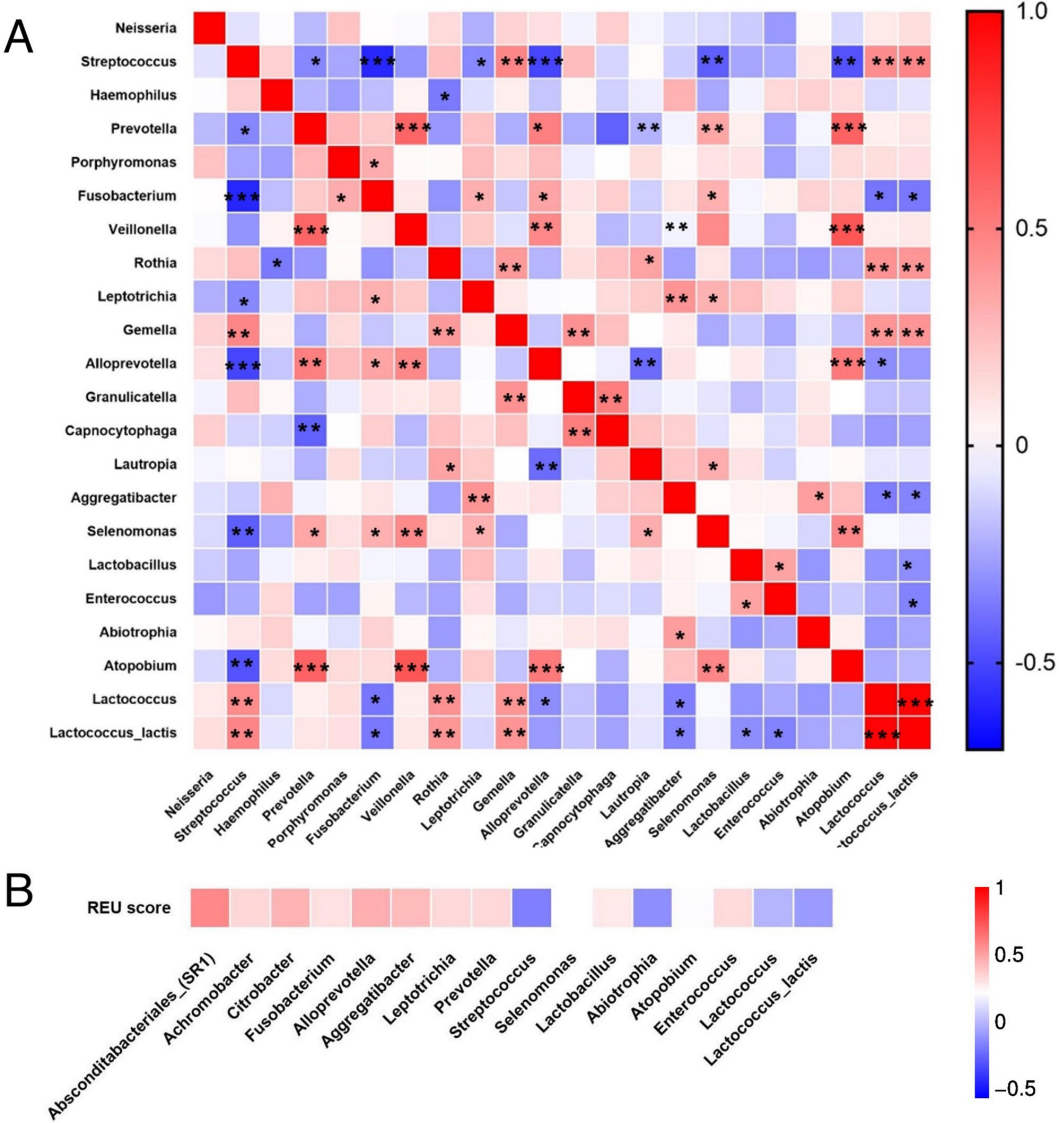
Relationship of certain bacteria in saliva sample (**A**) Heatmap showing correlation between lactic acid bacteria (LAB) and other bacterial genera. *P < 0.05, **P < 0.01, ***P < 0.001 (**B**) Correlation between key bacteria and REU score, which is indicative of oral lichen planus disease severity

Further, we investigated the co-occurrence of LAB and other bacterial genera in both groups. *Lactococcus* and *Lactococcus lactis* were significantly negatively related with *Fusobacterium* (ρ=-0.377, *P* = 0.013; ρ=-0.368, *P* = 0.015). Moreover, *Aggregatibacter* (ρ=-0.352, *P* = 0.021; ρ=-0.336, *P* = 0.028) showed similar relevance. *Streptococcus* was significantly negatively correlated with *Fusobacterium* (ρ=-0.594, *P* < 0.001), *Alloprevotella* (ρ=-0.510, *P* < 0.001), *Prevotella* (ρ=-0.331, *P* = 0.030), and *Leptotrichia* (ρ=-0.329, *P* = 0.031). However, *Gemella* was positively associated with *Streptococcus* (ρ = 0.470, *P* = 0.001); (Fig. 5A; Appendix 1).

### Relationship between LAB and clinical features of OLP patients

The association between LAB and other key microbes (the differential flora between OLP and HC or LAB related flora mentioned before) with OLP disease severity was analyzed using Spearman’s rank correlation coefficient. LAB, namely *Streptococcus*, *Lactococcus*, and *Lactococcus lactis*, were negatively correlated with REU score. However, the non-LAB genera, *Fusobacterium*, *Alloprevotella*, *Aggregatibacter*, *Leptotrichia*, and *Prevotella* were positively correlated with REU score (Fig. 5B).

## Discussion

In the present study, we elucidated that the alpha diversity of oral microbiota of both OLP and HC groups did not exhibit any significant differences, which is in accordance with the results of previous studies [13, 24]. Moreover, despite the overlap as per the PCoA plot between the microbiota composition of both groups, separation in beta diversity was observed; the microorganism distribution in the OLP group was more heterogeneous than in the HC group.

*Streptococcus* is the most commonly reported LAB, and its abundance has been reported to significantly decrease in OLP patients [10, 12, 13]. In a previous study, the abundance of *Streptococcus salivarius* was lower in OLP patients than in HC participants, and the supplementation of the bacterium in diet as a probiotic alleviated OLP lesions [25]. This could be because of its ability to inhibit NF-κB pathway activation, resulting in downregulation of innate immunity including inflammatory response of epithelial cells. While others like *Streptococcus pyogenes*, *Streptococcus agalactiae* and *Streptococcus pneumoniae* may be pathogens, even in OLP. In contrast, *Streptococcus intermedius* and *Streptococcus oralis* are considered potential pathogens in OLP patients, but the underlying mechanisms of their pathogenic activity are unclear [26]. Therefore, the abundance of different species of the *Streptococcus* in oral microbiota of OLP patients should be investigated in future studies.

To the best of our best knowledge, this is the first study to establish a relationship between LAB and OLP. LAB are a clade of gram-positive, catalase-negative, acid-fast bacteria, and *Streptococcus*, *Lactobacillus*, *Lactococcus*, *Leuconostoc*, and *Pediococcus* are the key members of LAB. In humans, they are a part of the oral microbiota. They share metabolic and physiological characteristics, mainly the production of lactic acid from sugars. Furthermore, some LAB species produce antimicrobial peptides known as bacteriocins; they also produce bioactive peptides with anticancer effect that are more effective at higher concentrations [27]. Numerous studies have reported anti-inflammatory and homeostatic effects of LAB, and they have been used to treat and prevent immune and inflammatory disorders, such as atopic dermatitis, inflammatory bowel disease, and multiple sclerosis [28, 29].

In the present study, at the genus level, only *Lactococcus* exhibited extremely decreased abundance in OLP patients. *Lactococcus* and *Streptococcus* were negatively correlated with OLP disease severity (REU score), suggesting that lower abundance of certain LAB species may lead to the deterioration of oral condition in OLP patients. LAB can modulate the composition of microbiota, by increasing the abundance of beneficial bacteria and decreasing that of harmful bacteria [30]. This is in accordance with our results that putative harmful bacteria exhibited negative correlation with LAB and positive correlation with disease severity (REU score).

Moreover, the abundance of *Lactococcus lactis* was lower in OLP patients than in HC participants. Correlation analysis revealed that higher abundance of *Lactococcus lactis* suggests alleviation in OLP. A previous study reported that higher abundance of *Lactococcus lactis* reduces inflammatory cytokine levels and protects against intestinal barrier damage in mice [31]; they are able to selectively degrade proinflammatory cytokines in inflamed intestinal tissue [32], suggesting that they have the potential to protect individuals from OLP lesions. Furthermore, *Lactococcus lactis* secrete lactocepins, bacterial enzymes, which can degrade other bacteria. Lipopeptides derived from *Lactococcus lactis*, lactococcin Gb acted to inhibit certain infection like SARS-CoV-2 [33]. *Lactococcus lactis LB 1022* exhibited nitric oxide (NO) suppression and increased the concentration of short-chain fatty acids (SCFAs) [34]. It has been found that NO level in the saliva and serum of OLP patients was significantly increased compared with HC [35]. This kind of oxidative stress damage may disrupt cellular proteins, DNA, lipids, and activate cellular immunity, contributing to pathogenesis of OLP [36]. On the other hand, SCFAs contribute to improving mucosa barrier damage in intestine of rat [37]. Shortage of *Lactococcus lactis* might produce less SCFAs, weakening capacity to repairing damaged mucosa barrier, which was believed to be related to the development of OLP [38]. An inverse statistical correlation was found between *Lactococcus lactis* and the putative harmful bacteria *Fusobacterium* and *Aggregatibacter*. However, the interaction between them should be further investigated in vivo and in vitro. This provides new insight into the potential of *Lactococcus lactis* as an adjunctive medication for OLP patients.

Moreover, this study elucidated that the abundance of opportunistic pathogens like *Citrobacter* increased in OLP patients compared with that in HC participants, and *Citrobacter* was positively related with severity of OLP. Similarly, in a previous study, higher abundance of *Citrobacter freundii* was associated with increased epithelial damage [39]. Perhaps it plays an important role in the progression of OLP disease even may play a part in its malignancy. *Citrobacter* was found to be one of the main microbes isolated from the oral squamous cell carcinoma sites [40], and supplementation of diet with LAB may reverse this phenomenon. As per animal studies, *Citrobacter rodentium* is a pathogen that can cause mucosa inflammation. In contrast, probiotic strains *Lactobacillus reuteri* and *Lactobacillus acidophilus* can relieve the severity of *Citrobacter rodentium* infections [41, 42]. However, longitudinal studies are required to establish whether *Citrobacter* can be used an indicator of OLP deterioration.

Therefore, in the present study, we elucidated that the decrease in the abundance of LAB could play a role in the onset or progression of OLP. However, 16 S rRNA sequencing is not suitable for identification at the species level, and other precise techniques should be developed to investigate the role of specific species in the pathogenesis of OLP; this could also help in the development of a probiotic-based treatment strategy for OLP.

### Electronic supplementary material

Below is the link to the electronic supplementary material.

Supplementary Material 1

## Data Availability

Sequence data that support the findings of the study are available in the NCBI Sequence Read Archive (SRA) repository under the BioProject ID PRJNA1049117. (https://www.ncbi.nlm.nih.gov/bioproject/PRJNA1049117).

## Abbreviations

OLP: Oral lichen planus
HC: Healthy control
LAB: Lactic acid bacteria
PD: Probing depth
REU: Reticular/hyperkeratotic, erosive/erythematous, ulcerative
PCR: Polymerase chain reaction
ASV: Amplicon sequence variant
PCoA: Principal coordinates analysis
LefSe: Linear discriminant analysis effect size
LDA: Linear discriminant analysis
SD: Standard deviation
