# Supplementary material for: Potential role of salivary lactic acid bacteria in pathogenesis of oral lichen planus
Source: BMC Microbiol. 2024 Jun 7;24:197. doi: 10.1186/s12866-024-03350-0 (PMC11157935; doi:10.1186/s12866-024-03350-0)
Supplement: Supplementary file 1 — Supplementary Material 1 [file 12866_2024_3350_MOESM1_ESM.docx]

| Appendix 1. correlation between lactic acid bacteria (LAB) and other bacterial genera | | | |
| --- | --- | --- | --- |
| Bacterium 1 | Bacterium 2 | ρ | *P* |
| *Lactococcus* | *Streptococcus* | 0.446 | 0.003 |
| *Lactococcus* | *Fusobacterium* | -0.377 | 0.013 |
| *Lactococcus* | *Rothia* | 0.422 | 0.005 |
| *Lactococcus* | *Gemella* | 0.404 | 0.007 |
| *Lactococcus* | *Alloprevotella* | -0.312 | 0.041 |
| *Lactococcus* | *Aggregatibacter* | -0.352 | 0.021 |
| *Lactococcus* | *Lactococcus_lactis* | 0.981 | 0.000 |
| *Lactococcus_lactis* | *Streptococcus* | 0.472 | 0.001 |
| *Lactococcus_lactis* | *Fusobacterium* | -0.368 | 0.015 |
| *Lactococcus_lactis* | *Rothia* | 0.407 | 0.007 |
| *Lactococcus_lactis* | *Gemella* | 0.412 | 0.006 |
| *Lactococcus_lactis* | *Aggregatibacter* | -0.336 | 0.028 |
| *Lactococcus_lactis* | *Lactobacillus* | -0.312 | 0.042 |
| *Lactococcus_lactis* | *Enterococcus* | -0.341 | 0.025 |
| *Atopobium* | *Streptococcus* | -0.473 | 0.001 |
| *Atopobium* | *Prevotella* | 0.608 | 0.000 |
| *Atopobium* | *Veillonella* | 0.662 | 0.000 |
| *Atopobium* | *Alloprevotella* | 0.521 | 0.000 |
| *Atopobium* | *Selenomonas* | 0.469 | 0.002 |
| *Abiotrophia* | *Aggregatibacter* | 0.380 | 0.012 |
| *Enterococcus* | *Lactobacillus* | 0.355 | 0.019 |
| *Selenomonas* | *Streptococcus* | -0.441 | 0.003 |
| *Selenomonas* | *Prevotella* | 0.348 | 0.022 |
| *Selenomonas* | *Fusobacterium* | 0.302 | 0.049 |
| *Selenomonas* | *Veillonella* | 0.452 | 0.002 |
| *Selenomonas* | *Leptotrichia* | 0.301 | 0.050 |
| *Selenomonas* | *Lautropia* | 0.314 | 0.040 |
| *Aggregatibacter* | *Leptotrichia* | 0.415 | 0.006 |
| *Lautropia* | *Rothia* | 0.356 | 0.019 |
| *Lautropia* | *Alloprevotella* | -0.404 | 0.007 |
| *Capnocytophaga* | *Prevotella* | -0.432 | 0.004 |
| *Capnocytophaga* | *Granulicatella* | 0.502 | 0.001 |
| *Granulicatella* | *Gemella* | 0.426 | 0.004 |
| *Alloprevotella* | *Streptococcus* | -0.510 | 0.000 |
| *Alloprevotella* | *Prevotella* | 0.495 | 0.001 |
| *Alloprevotella* | *Fusobacterium* | 0.352 | 0.020 |
| *Alloprevotella* | *Veillonella* | 0.465 | 0.002 |
| *Gemella* | *Streptococcus* | 0.470 | 0.001 |
| *Gemella* | *Rothia* | 0.395 | 0.009 |
| *Leptotrichia* | *Streptococcus* | -0.329 | 0.031 |
| *Leptotrichia* | *Fusobacterium* | 0.309 | 0.044 |
| *Rothia* | *Haemophilus* | -0.365 | 0.016 |
| *Veillonella* | *Prevotella* | 0.598 | 0.000 |
| *Fusobacterium* | *Streptococcus* | -0.594 | 0.000 |
| *Fusobacterium* | *Porphyromonas* | 0.324 | 0.034 |
| *Prevotella* | *Streptococcus* | -0.331 | 0.030 |
